# Supplementary material for: Self-Assembled Amphiphilic Chitosan Nanomicelles: Synthesis, Characterization and Antibacterial Activity
Source: Biomolecules. 2023 Oct 30;13(11):1595. doi: 10.3390/biom13111595 (PMC10669896; doi:10.3390/biom13111595)
Supplement: Supplementary file 1 [file biomolecules-13-01595-s001.zip › biomolecules-2650122-supplementary.pdf]

Figure captions

Fig. S1. Synthesis route of CS-DA.

Fig. S2. Preparation of the chitosan functionalized with deoxycholic acid and succinic anhydride (CS-DA-SA).

Fig. S3. (A) FTIR spectra of CS, DA, SA, CS-DA, and CS-DA-SA; (B) ^{1}H NMR spectra of CS, DA, SA, CS-DA, and CS-DA-SA; (C) XRD spectra of CS, CS-DA, and CS-DA-SA.

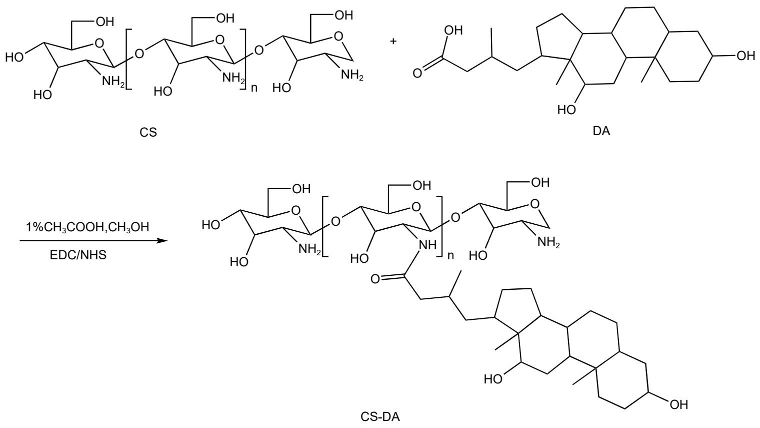

Fig. S1. Synthesis route of CS-DA

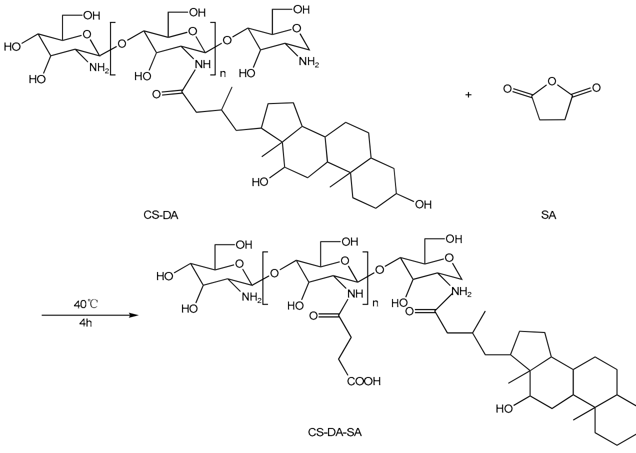

Fig. S2. Preparation of the chitosan functionalized with deoxycholic acid and succinic anhydride (CS-DA-SA).

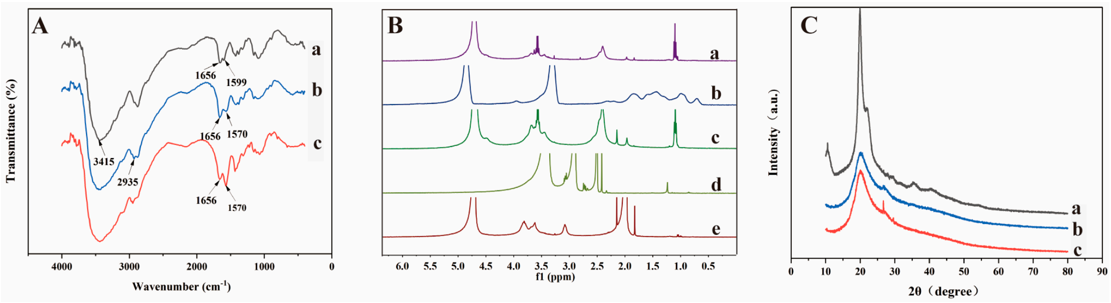

Fig. S3. (A) FTIR spectra of CS, DA, SA, CS-DA, and CS-DA-SA; (B) ^{1}H NMR spectra of CS, DA, SA, CS-DA, and CS-DA-SA; (C) XRD spectra of CS, CS-DA, and CS-DA-SA.

Synthesis and characterization of chitosan derivatives

The chitosan derivatives (CS-DA-SA) were successfully prepared (Fig.S3). The structure of CS-DA-SA was evaluated and verified by FTIR (Fig.S3A) and ^{1}H NMR. For the unmodified chitosan, the absorption band at 3415 cm^{-1} was attributed to stretching vibrations of -OH and –NH_2, while the peak at around 2870 cm^{-1} was associated with -CH vibrations. The peaks centred at 1651 cm^{-1},1594 and 1082 cm^{-1} were attributed to -C=O, -N-H, and acetyl vibrations. All these bands are characteristic of chitosan FTIR spectra. The absorption peak at 1708 cm^{-1} represented the carbonyl moiety of DA. Compared with the infrared spectra of chitosan, the peaks of amide I and amide II in the CS-DA spectrum showed red shift, indicating that the amide group was formed on the primary amine of chitosan. In addition, the peak was also enhanced at 2935 cm^{-1}, which represented the stretching vibration absorption peak of methylene, indicating the successful graft of deoxycholic acid into methylene. Compared with the infrared spectra of CS-DA, the band of amide II was significantly enhanced at 1570 cm^{-1}, which also indicated the amide group was formed at C_2-NH_2. In conclusion, amphiphilic chitosan was successfully synthesized by introducing hydrophilic and hydrophobic groups into the C_2-NH_2 position of chitosan.

The derivatives of chitosan were also successfully prepared and polymer behaviors were evaluated by ^{1}H NMR signal decay profiles (Fig.S3B). In the deoxycholic acid spectrogram, the -CH_3 and -CH_2 characteristic proton peaks appear at 0.5-2.5 ppm [1]. We can see from the spectrogram that 2.12-2.71 ppm in the spectrogram of succinic anhydride is the proton signal of methylene on the succinyl group [2]. It was found that the characteristic proton peaks of deoxycholic acid at 0.5-2.5 ppm and succinic anhydride at 2.12-2.71 ppm appeared on the CS-DA-SA spectra, respectively, which indicated that the amphiphilic chitosan was successfully prepared.

XRD spectra were also used to verify the structures of the derivatives (Fig.S3C). Two-dimensional chitosan crystals produce strong diffraction peaks at 15°-25°, which is a crystallization feature caused by hydrogen bonds between amino groups and

hydroxyl groups [3]. Compared with CS， the CS-DA curve was relatively flat and the diffraction curve peak area was wider, while the characteristic peak intensity was lower. It indicated that the free amino group was reduced and the natural crystal form of chitosan was destroyed, which means that DA was grafted to the amino group of chitosan. As seen from the CS-DA-SA curve, the intensity of the diffraction peak was weakened than the CS-DA curve. This was because SA was grafted to CS-DA and the substitution reaction of SA led to the gradual reduction of free amino group and internal hydrogen bond, resulting in reduced crystallinity [4]. These findings demonstrated the successful preparation of the target product CS-DA-SA.

References

1. Wu, M.; Guo, K.; Dong, H.; Zeng, R.; Tu, M.; Zhao, J. In vitro drug release and biological evaluation of biomimetic polymeric micelles self-assembled from amphiphilic deoxycholic acid-phosphorylcholine-chitosan conjugate. *Materials Science & Engineering C-Materials for Biological Applications* **2014**, *45*, 162-169. http://dx.doi.org/10.1016/j.msec.2014.09.008
2. Niu, X.; Zhu, L.; Xi, L.; Guo, L.; Wang, H. An antimicrobial agent prepared by N-succinyl chitosan immobilized lysozyme and its application in strawberry preservation. *Food Control* **2020**, *108*, 106829. http://dx.doi.org/10.1016/j.foodcont.2019.106829
3. Zidan, T.A.; Abdelhamid, A.E.; Zaki, E.G. N-Aminorhodanine modified chitosan hydrogel for antibacterial and copper ions removal from aqueous solutions. *International Journal of Biological Macromolecules* **2020**, 32-42. http://dx.doi.org/10.1016/j.ijbiomac.2020.04.180
4. Han, X.; Zheng, Z.; Yu, C.; Deng, Y.; Ye, Q.; Niu, F.; Chen, Q.; Pan, W.; Wang, Y. Preparation, characterization and antibacterial activity of new ionized chitosan. *Carbohydrate Polymers* **2022**, *290*, 119490. http://dx.doi.org/10.1016/j.carbpol.2022.119490
